# *In vivo* imaging demonstrates dendritic spine stabilization by SynCAM 1

**DOI:** 10.1038/srep24241

**Published:** 2016-04-07

**Authors:** Nils Körber, Valentin Stein

**Affiliations:** 1 Institute of Physiology II, Medical Faculty, University Bonn, Bonn, Germany

## Abstract

Formation and stability of synapses are required for proper brain function. While it is well established that synaptic adhesion molecules are important regulators of synapse formation, their specific role during different phases of synapse development remains unclear. To investigate the function of the synaptic cell adhesion molecule SynCAM 1 in the formation, stability, and maintenance of spines we used 2-photon *in vivo* imaging to follow individual spines over a long period of time. In SynCAM 1 knockout mice the survival rate of existing spines was reduced and fewer filopodia-like structures were converted into stable spines. SynCAM 1^flag^ overexpression resulted in more stable spines and fewer filopodia-like structures. When SynCAM 1^flag^ overexpression is turned on the spine density rapidly increases within a few days. Interestingly, the spine density stayed at an elevated level when SynCAM 1^flag^ overexpression was turned off. Our data indicate that the SynCAM 1 induced altered spine density is not caused by the formation of newly emerging protrusions, instead SynCAM 1 stabilizes nascent synaptic contacts which promotes their maturation. Concomitant with the synaptic stabilization, SynCAM 1 generally prolongs the lifetime of spines. In summary, we demonstrate that SynCAM 1 is a key regulator of spine stability.

Dynamic changes of synaptic spines are associated with cognitive functions such as learning and memory. The formation and maintenance of synaptic spines is critically dependent on synaptic adhesion molecules^1^. According to the so called filopodia model, synapse formation can be divided into three main phases^2^,^3^ in which synaptic cell adhesion molecules are involved. First, a thin protrusion of the dendrite emerges and seeks contact to a nearby axon requiring target cell recognition. Importantly, this first phase is likely preceded by assembling a molecular machinery required to realize the structural changes of the dendrite to form a protrusion. In the second phase, the initial contact is stabilized by physically linking the membranes of the two different cells by the extracellular interaction of synaptic adhesion molecules. In parallel, further proteins are recruited to the developing pre- and postsynapse mediated by intracellular protein-protein interaction domains of synaptic adhesion molecules. In the third phase synaptic adhesion molecules maintain synaptic connections by extracellular interaction and providing intracellular binding sites for other synaptic proteins. Although synaptic adhesion molecules have been intensely studied, it is still unclear at which stage of the spine lifecycle the different synaptic adhesion molecules operate. Various synaptic adhesion molecules induce synapse formation when overexpressed in heterologous cells^4^,^5^; however, this might not represent their physiological function, as synaptic adhesion molecules also function in later phases of synapse development and maintenance.

Members of the neuroligin, SynCAM and EphB receptor families are involved in the morphologic and functional differentiation of synapses^4^,^5^,^6^,^7^,^8^. Synapse disorganization and imbalanced neuronal excitation and inhibition lead to neurological disorders^9^. Consistent with the physiological relevance of synapse-organizing molecules, neuroligin, neurexin, SynCAM 1 and cadherins have been linked to neurological disorders such as Alzheimer’s disease, autism spectrum disorders and schizophrenia^10^,^11^,^12^,^13^,^14^,^15^.

Here we focus on the function of SynCAM 1 in spine formation and maintenance. SynCAM 1 belongs to the immunoglobulin superfamily (IgSF). The SynCAM family comprises four members in mammals (SynCAM 1–4)^16^ that are localized at pre- and postsynaptic terminals. In the central nervous system (CNS) mainly the heterophilic interaction of SynCAM 1 and SynCAM 2 occurs^17^,^18^. We reported earlier that overexpression of SynCAM 1^flag^ leads to an increase in synapse density and the loss of SynCAM 1 causes a decrease in synapse density in hippocampal CA1 pyramidal cells^19^. Overexpressing or deleting SynCAM 1 had neither an effect on presynaptic release nor on postsynaptic receptors; however, long-term depression (LTD) was impaired in SynCAM 1 overexpressing mice and facilitated in SynCAM 1 knockout animals. These findings indicate that SynCAM 1 rather acts on the structural properties of the synapse.

Identifying the physiological role of a synaptic adhesion molecule is still a challenging question. The time domains of synapse formation and maturation are quite diverse; while forming a spine might take some minutes to hours, spines can be stable for several months. To cover the extended time domain we tracked individual spines in living animals by two-photon *in vivo* microscopy of layer V pyramidal neurons in the visual cortex. We used Thy1-GFP mice that express the green fluorescent protein (GFP) in a sparse subset of cortical neurons^20^.

Here, we demonstrate that SynCAM 1 directly stabilizes newly formed contacts, thereby impacting on the maturation state of spines. Furthermore, SynCAM 1 improves the stability of mature spines. Inducing the overexpression of SynCAM 1 increases the spine density within a few days. Interestingly, the spine density does not rapidly return to control levels after turning the overexpression of SynCAM 1^flag^ off. However, our data do not support the idea, that SynCAM 1 directly induces the formation of nascent protrusions. In summary, we show that SynCAM 1 is a key regulator of the spine stability.

## Results

To study the effect of SynCAM 1 on spine dynamics, we employed *in-vivo* two photon microscopy to follow the fate of individual spines over a long period of time in adult SynCAM 1 knockout (KO) mice completely lacking SynCAM 1^21^ and adult SynCAM 1^flag^ overexpressing mice (OE)^19^; SynCAM 1^flag^ expression is driven by a tet-off system under the control of the CaMKII promotor allowing to suppress ectopic overexpression^22^. Both lines were bread to the GFP-M line to obtain sparsely labeled individual cortical pyramidal neurons^20^.

### Spines in SynCAM 1 knockout mice are less stable

**Figure 1:**
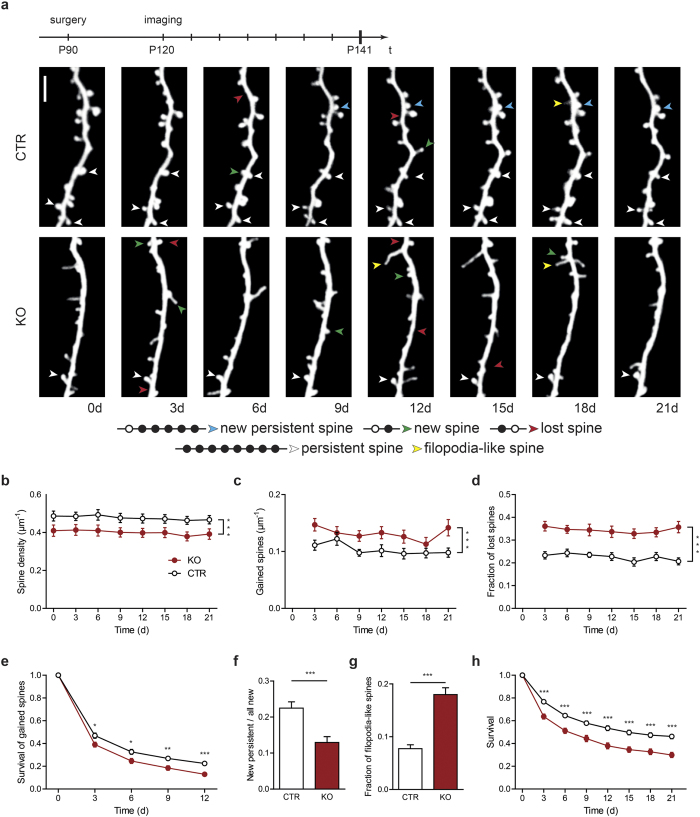
Loss of SynCAM 1 decreases spine stability and impairs *de novo* spine stabilization. (**a**) Timeline of the experimental procedure (on top). Representative *in vivo* images at eight different time points of a dendritic branch from a layer V pyramidal neuron of a control (top row) and a knockout mouse (bottom row), scale bar 5 μm. Bottom, categories for spine definition, open circle represent the absence of the spine, closed circle represents the presence of the spine. (**b**) The spine density of SynCAM 1 deficient mice (red circles, KO) compared to control mice (open circles, CTR) is reduced. (**c**) Spine gain is elevated in SynCAM 1 knockout mice compared to control mice. (**d**) Spine loss is strongly increased in SynCAM 1 knockout mice compared to control mice. (**e**) Survival rate of spines which newly emerged is lower in SynCAM 1 knockout mice. (**f**) Fraction of new persistent spines of all new spines is reduced in knockout animals. (**g**) More filopodia-like structures are present in SynCAM 1 knockout mice. (**h**) Survival rate of spine present at the first time point. Data were presented as means ± SEM, *p < 0.05, **p < 0.001, ***p < 0.0001, and obtained from CTR: 33 dendrites, 5 animals; KO: 29 dendrites, 5 animals.

First, we analyzed the spine density of layer V pyramidal neurons, which was significantly reduced in adult SynCAM 1 KO mice compared to control (CTR) littermates (Fig. 1a,b; CTR 0.48 ± 0.03 spines/μm, KO 0.40 ± 0.02 spines/μm, N = 33 dendrites from 5 animals (CTR) and N = 29 dendrites from 5 animals (KO), p < 0.0001, paired t-test of the means, see methods). Expectedly, the spine density was stable for the period of three weeks.

To decipher the underlying mechanism leading to the reduction in spine density, we counted the number of gained spines at each time point. Importantly, we assume that the spine gain depends on the length of dendrite studied, while the spine loss depends on the number of existing spines; hence we calculated spine gain as spines per length and spine loss as fraction of lost spines of initially present spines. The number of gained spines is increased in SynCAM 1 KO mice compared to CTR littermates (Fig. 1c; CTR 0.10 ± 0.01 spines/μm per 3 days, KO 0.13 ± 0.01 spines/μm per 3 days, N = 33 dendrites from 5 animals (CTR) and N = 29 dendrites from 5 animals (KO), p = 0.0006, paired t-test of the means). The increased fraction of lost spines (Fig. 1d; CTR 23 ± 2% per 3 days, KO 35 ± 2% per 3 days, N = 33 dendrites from 5 animals (CTR) and N = 29 dendrites from 5 animals (KO), p < 0.0001, paired t-test of the means) indicates that spines in SynCAM 1 KO mice are less stable. Therefore, we analyzed the survival of newly gained spines, i.e. spines that were detected for the first time at time point 3d, 6d, or 9d and followed them for the next 12 days (Fig. 1e; after 3 days: CTR 47 ± 2%, KO 39 ± 2%, p = 0.015; after 12 days: CTR 22 ± 2%, KO 13 ± 2%, p = 0.0002; N = 33 dendrites from 5 animals (CTR) and N = 29 dendrites from 5 animals (KO), unpaired t-test). This analysis reveals a biphasic loss of newly gained spines, with an initial fast component where more than 50% of the new spines are lost in the first 3 days and a slower component for the following days. Both rates are larger in SynCAM 1 KO animals indicating that fewer newly formed spines are converted into longer lasting spines. This is further reflected in the reduced ratio of new persistent spines to all new spines (Fig. 1f; CTR 22 ± 2%, KO 13 ± 2%; N = 33 dendrites from 5 animals (CTR) and N = 29 dendrites from 5 animals (KO), p = 0.0002, unpaired t-test). If fewer nascent spines are converted into spines we might see more immature structures. Several studies suggest that immature/young spines are thin dendritic protrusions (filopodia-like spines) and more mature/stable spines possess a bulbous head^3^,^23^,^24^; therefore, we should see an altered ratio of these filopodia-like spines to spines which have a bulbous head. Indeed, the fraction of filopodia-like spines is more than doubled in SynCAM 1 KO mice (Fig. 1g; CTR: 8 ± 1%, KO: 18 ± 2%, N = 33 dendrites from 5 animals (CTR) and N = 29 dendrites from 5 animals (KO), p < 0.0001, unpaired t-test). Finally, we determined the survival ratio of each spine that existed at the first time point (Fig. 1h; after 3 days: CTR 77 ± 2%, KO 64 ± 2%, p < 0.0001; after 21 days: CTR 46 ± 2%, KO 30 ± 2%, p < 0.0001; N = 33 dendrites from 5 animals (CTR) and N = 29 dendrites from 5 animals (KO), unpaired t-test). Most importantly, preexisting spines have a lower rate of survival in SynCAM 1 deficient animals compared to control animals, indicating that SynCAM 1 not only promotes the transition from nascent spines to mature spines, but improves the stability of persistent spines.

Taken together, the lower spine density in SynCAM 1 KO mice is caused by two factors, nascent spines mature less frequently into stable spines and spines in general exhibit a shorter survival time showing that SynCAM 1 is involved in spine maturation and stability, but does not promote the initial formation of nascent protrusions.

### SynCAM 1^flag^ overexpression stabilizes spines

**Figure 2:**
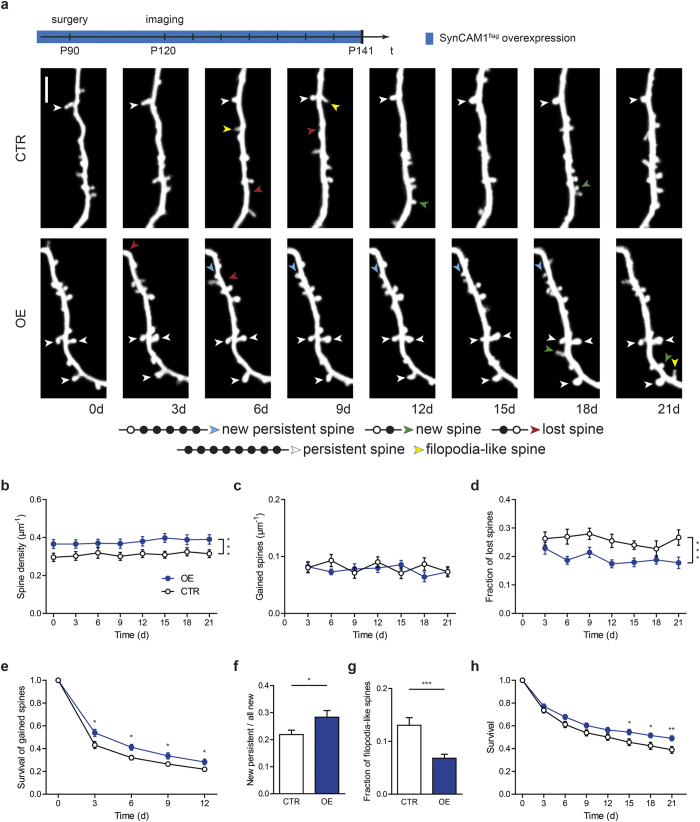
Constant overexpression of SynCAM 1^flag^ increases stability and *de novo* spine stabilization. (**a**) Timeline of the experimental procedure (on top). Representative *in vivo* images at eight different time points of a dendritic branch from a layer V pyramidal neuron of a control (top row) and a SynCAM 1^flag^ overexpressing mouse (bottom row), scale bar 5 μm. Bottom, categories for spine definition, open circle represent the absence of the spine, closed circle represents the presence of the spine. (**b**) The spine density of SynCAM 1^flag^ overexpressing mice (blue circles, OE) compared to control mice (open circles, CTR) is increased. (**c**) No difference in spine gain is observed between SynCAM 1^flag^ overexpressing mice and control mice. (**d**) Spine loss is decreased in SynCAM 1^flag^ overexpressing mice compared to control mice. (**e**) Survival rate of spines which newly emerged is higher in SynCAM 1^flag^ overexpressing mice. (**f**) Fraction of new persistent spines of all new spines is increased in SynCAM 1^flag^ overexpressing animals. (**g**) Fewer filopodia-like structures are found in SynCAM 1^flag^ overexpressing mice. (**h**) Survival rate of spine present at the first time point. Data were presented as means ± SEM, *p < 0.05, **p < 0.001, ***p < 0.0001, and obtained from CTR: 21 dendrites, 7 animals; OE: 27 dendrites, 7 animals.

Next, we asked whether overexpressing SynCAM 1^flag^ has the opposite effect of completely removing SynCAM 1 on spine stability. Mice overexpressing SynCAM 1^flag^ had an increased spine density (Fig. 2a,b; CTR 0.31 ± 0.02 spines/μm, OE 0.38 ± 0.02 spines/μm, N = 21 dendrites from 7 animals (CTR) and N = 27 dendrites from 7 animals (OE), p < 0.0001, paired t-test of the means), contrasting our results of SynCAM 1 knockout mice. We noted a higher spine density in controls of the KO mice compared to the controls of the SynCAM 1^flag^ overexpressing mice (Figs 1a and 2a). This reflects the different genetic backgrounds of the KO and transgenic mouse strains used in this study and was already reported in our earlier study using the same mouse strains^19^. We performed the same analysis as for SynCAM 1 KO described in Fig. 1. SynCAM 1^flag^ overexpression did not lead to an increased spine gain (Fig. 2c; CTR 0.08 ± 0.01 spines/μm per 3 days, OE 0.08 ± 0.01 spines/μm per 3 days, N = 21 dendrites from 7 animals (CTR) and N = 27 dendrites from 7 animals (OE), p = 0.45, paired t-test of the means), again suggesting that SynCAM 1 is not involved in the formation of nascent protrusions. However, the reduced fraction of lost spines indicates that spines are more stable in SynCAM 1^flag^ mice compared to control littermates (Fig. 2d; CTR 26 ± 2% per 3 days, OE 19 ± 2% per 3 days, N = 21 dendrites from 7 animals (CTR) and N = 27 dendrites from 7 animals (OE), p = 0.0002, paired t-test of the means). Indeed, comparing the survival of newly-gained spines reveals that additional SynCAM 1 increases the survival of gained spines (Fig. 2e; after 3 days: CTR 43 ± 3%, OE 54 ± 3%, p = 0.019; after 12 days: CTR 22 ± 2%, OE 28 ± 2%, p = 0.047, p = 0.0002; N = 21 dendrites from 7 animals (CTR) and N = 27 dendrites from 7 animals (OE), unpaired t-test). This is also reflected in the increased ratio of new persistent spine to all new spines (Fig. 2f; CTR 22 ± 2%, OE 28 ± 2%, p = 0.0002; N = 21 dendrites from 7 animals (CTR) and N = 27 dendrites from 7 animals (OE), unpaired t-test). In analogy to the analysis of SynCAM 1 KO, a decreased fraction of filopodia-like spines in OE mice suggests, that immature structures are converted with a higher likelihood into mature spines (Fig. 2g; CTR: 13 ± 2%, OE: 7 ± 1%, p < 0.0001, N = 17 dendrites from 6 animals (CTR) and N = 19 dendrites from 6 animals (OE), unpaired t-test). Finally, we determined the survival rate of each spine that existed at the first time point (Fig. 2h; after 3 days: CTR 74 ± 2%, OE 77 ± 2%, p = 0.28; after 21 days: CTR 39 ± 3%, OE 49 ± 2%, p = 0.0075; N = 21 dendrites from 7 animals (CTR) and N = 27 dendrites from 7 animals (OE), unpaired t-test). General survival of spines is significantly increased in OE mice.

In summary, overexpressing SynCAM 1^flag^ does not increase the number of new spines, but stabilizes new as well as existing spines and promotes spine maturation, which in turn leads to the increased spine density.

### Induction of SynCAM 1^flag^ overexpression alters spine density

**Figure 3:**
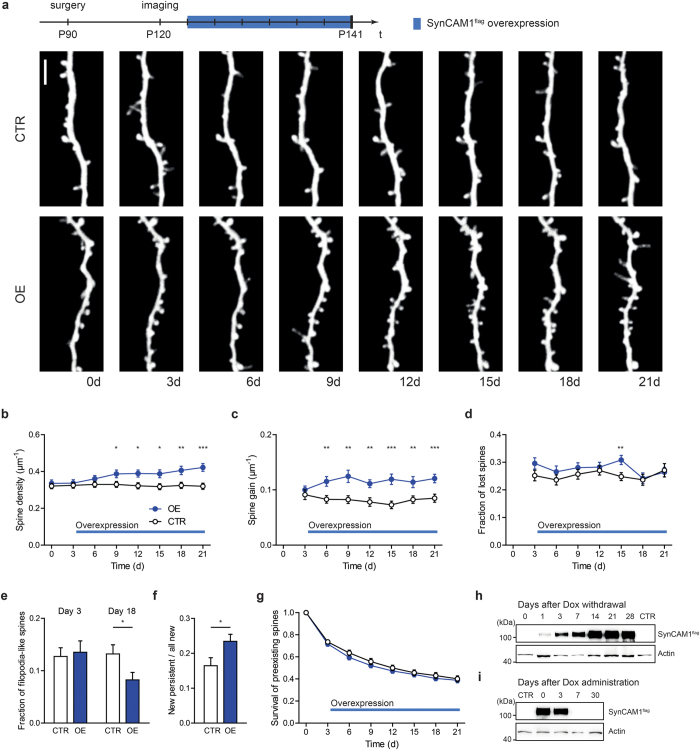
Induced overexpression of SynCAM 1^flag^ increases spine density. (**a**) Timeline of the experimental procedure (on top). Representative *in vivo* images at eight different time points of a dendritic branch from a layer V pyramidal neuron of a control (top row) and a SynCAM 1^flag^ overexpressing mouse in which SynCAM 1^flag^ overexpression was switched on after the 2^nd^ imaging session (bottom row), scale bar 5 μm. (**b**) The spine density of SynCAM 1^flag^ overexpressing (blue circles, OE) mice compared to control mice (open circles, CTR) starts to increase as soon as overexpression is turned on. (**c**) An elevated spine gain is observed in SynCAM 1^flag^ overexpressing mice. (**d**) No difference was observed in spine loss. (**e**) Fewer filopodia-like structures are found after the induction of SynCAM 1^flag^ overexpression. (**f**) Fraction of new persistent spines of all new spines is increased in SynCAM 1^flag^ overexpressing animals. (**g**) SynCAM 1^flag^ overexpression does not affect the survival of spines that existed before SynCAM 1^flag^ overexpression was turned on. (**h**) Induction of SynCAM 1^flag^ overexpression is detectable already after one day of doxycycline withdrawal and reaches saturation after two weeks. (**i**) Doxycycline administration completely suppresses SynCAM 1^flag^ overexpression within seven days. Data were presented as means ± SEM, *p < 0.05, **p < 0.001, ***p < 0.0001, and obtained from CTR: 32 dendrites, 8 animals; OE: 29 dendrites, 7 animals.

If SynCAM 1 is a robust regulator of spine stability, we would expect that dynamically changing SynCAM 1^flag^ expression rather quickly affects spine density. In this part we suppressed SynCAM 1^flag^ overexpression by doxycycline administration starting at E14 until the second imaging time point at around P120 (see Fig. 3a, time line on top). Only a few days after turning on SynCAM 1^flag^ overexpression the spine density started to continuously increase over the next 18 days (Fig. 3a,b; day 6: CTR 0.33 ± 0.02 spines/μm, OE 0.36 ± 0.02 spines/μm, p = 0.2; day 21: CTR 0.32 ± 0.02 spines/μm, OE 0.42 ± 0.02 spines/μm, p < 0.0003, N = 32 dendrites from 8 animals (CTR) and N = 29 dendrites from 7 animals (OE), unpaired t-test). In contrast to static conditions, we observed an increased spine gain when elevating SynCAM 1^flag^ (Fig. 3c; day 6: CTR 0.08 ± 0.01 spines/μm per 3 days, OE 0.11 ± 0.01 spines/μm per 3 days, p = 0.0021; day 21: CTR 0.08 ± 0.01 spines/μm per 3 days, OE 0.12 ± 0.01 spines/μm per 3 days, p = 0.0001, N = 32 dendrites from 8 animals (CTR) and N = 29 dendrites from 7 animals (OE), unpaired t-test) and no differences in spine loss (Fig. 3d; day 6: CTR 24 ± 2% per 3 days, OE 27 ± 2% per 3 days, p = 0.3; day 21: CTR 27 ± 2% per 3 days, OE 26 ± 2% per 3 days, p = 0.8, N = 32 dendrites from 8 animals (CTR) and N = 29 dendrites from 7 animals (OE), unpaired t-test). Importantly, the increase in spine gain starts as soon as overexpression of SynCAM 1^flag^ is turned on as shown by Western blot analysis (Fig. 3h).

The change in the fraction of filopodia-like spines after the induction of SynCAM 1^flag^ overexpression indicates that not more newly emerging structures are formed but that nascent protrusions mature at a higher likelihood (Fig. 3e; at day 3: CTR 13 ± 2%, OE 14 ± 2%, p = 0.77; N = 17 (CTR) and 18 (OE); at day 18: CTR 13 ± 2%, OE 8 ± 2%, p = 0.03; N = 17 dendrites from 6 animals (CTR) and N = 18 dendrites from 6 animals (OE), unpaired t-test). In addition, more spines newly formed at the beginning of SynCAM 1^flag^ overexpression (day 6–9) are persistent (Fig. 3f; CTR: 16 ± 2%, OE: 23 ± 2%, p < 0.027, N = 32 dendrites from 8 animals (CTR) and N = 29 dendrites from 7 animals (OE), unpaired t-test). Interestingly, SynCAM 1^flag^ did not affect the survival of preexisting spines, i.e. spines that were present before SynCAM 1^flag^ overexpression was turned on (Fig. 3g; after 3 days: CTR 74 ± 2%, OE 72 ± 2%, p = 0.54; after 21 days: CTR 40 ± 3%, OE 39 ± 2%, p = 0.71; N = 32 dendrites from 8 animals (CTR) and N = 29 dendrites from 7 animals (OE), unpaired t-test). Taken together, acutely turning on SynCAM 1^flag^ overexpression increases the stability of newly emerging spines, but does not stabilize preexisting spines.

**Figure 4:**
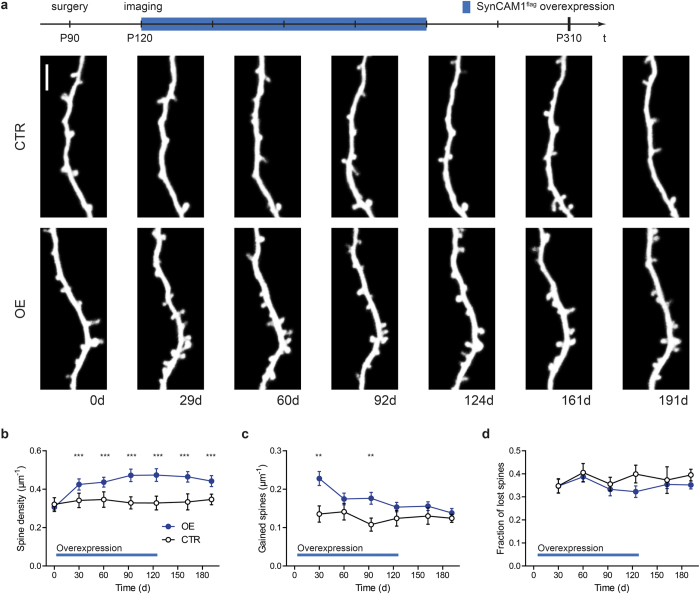
Long term overexpression of SynCAM 1^flag^ constantly increases the spine density. (**a**) Timeline of the experimental procedure (on top). Representative *in vivo* images at seven different time points of a dendritic branch from a layer V pyramidal neuron of a control (top row) and a SynCAM 1^flag^ overexpressing mouse in which SynCAM 1^flag^ overexpression was switched on after the first imaging session and switched off four months later (bottom row), scale bar 5 μm. (**b**) Spine densities analyzed monthly for SynCAM 1^flag^ overexpressing (blue circles, OE) and control (open circles, CTR) mice. After an initial increase the spine density stays constant at a higher level. (**c**) Temporarily, the spine gain is strongly increased but drops down to control levels after the first 30 days of SynCAM 1^flag^ overexpression. (**d**) No difference was observed in spine loss. Data were presented as means ± SEM, *p < 0.05, **p < 0.001, ***p < 0.0001, and obtained from CTR: 12 dendrites, 4 animals; OE: 20 dendrites, 5 animals.

After an extended period of overexpression the spine density should reach a new steady state. To test this idea, we overexpressed SynCAM 1^flag^ for four months taking images every 30 days (Fig. 4a); additionally, we tested the reversibility by turning off the overexpression after four months. Turning on SynCAM 1^flag^ overexpression led to an increase in spine density within the first 30 days reaching a new steady state thereafter (Fig. 4a,b; day 0: CTR 0.32 ± 0.03 spines/µm, OE 0.31 ± 0.02 spines/μm, day 30: CTR 0.34 ± 0.04 spines/µm, OE 0.42 ± 0.03 spines/μm, p < 0.0001 paired t-test; day 191: CTR 0.35 ± 0.03 spines/μm, OE 44 ± 0.03 spines/μm, p = 0.049, N = 12 from 4 animals (CTR) and N = 20 from 5 animals (OE), unpaired t-test). During the initial phase of increasing spine density the spine gain was strongly increased and returned back to baseline within the next months (Fig. 4c; day 30: CTR 0.14 ± 0.02 spines/μm per 30 days, OE 0.23 ± 0.02 spines/μm per 30 days, p = 0.0031; day 124: CTR 0.12 ± 0.02 spines/μm per 30 days, OE 0.15 ± 0.01 spines/μm per 30 days, p = 0.20, N = 12 from 4 animals (CTR) and N = 20 from 5 animals (OE), unpaired t-test). Meanwhile the spine loss is not affected (Fig. 4d; day 30: CTR 35 ± 3%, OE 35 ± 3%, p = 0.98; day 124: CTR 40 ± 4%, OE 32 ± 3%, p = 0.09, N = 12 from 4 animals (CTR) and N = 20 from 5 animals (OE), unpaired t-test). In summary, this confirms our hypothesis that acutely elevating SynCAM 1 levels transiently perturbs spine dynamics until a new equilibrium is reached.

Surprisingly, when we suppressed SynCAM 1^flag^ overexpression after four months the spine density stayed at an elevated level for the next two months (Fig. 4a,b). The persistent elevation of the spine density might indicate that other factors preserve the spine density. Importantly, Western blot analysis excludes that SynCAM 1^flag^ overexpression was not fully repressed by doxycycline administration (Fig. 3h).

## Discussion

Here we describe the effects of the synaptic adhesion molecule SynCAM 1 on spine dynamics studied in the living animal. Our data provide a new insight into the functional mechanisms of SynCAM 1. In general, SynCAM 1 regulates spine stability and maturation; however, SynCAM 1 does not directly induce the formation of nascent protrusions. In SynCAM 1 knockout mice fewer filopodia-like structures were converted into stable spines and the survival rate of preexisting spines was reduced. SynCAM 1^flag^ overexpression resulted in fewer filopodia-like structures and more stable spines. These effects result in a decreased spine density in SynCAM 1 KO mice and an increased spine density in SynCAM 1^flag^ overexpressing mice. The spine stabilizing action of SynCAM 1 is most directly seen when SynCAM 1^flag^ overexpression was turned on during the series of imaging sessions resulting in a rapid increase of the spine density within a few days. Interestingly, the spine density stayed at an elevated level when overexpression was turned off.

Generally, a synaptic adhesion molecule could affect three factors of spine formation and maintenance: 1. The number of emerging nascent protrusions 2. Conversion rate of nascent protrusions into stable dendritic spines. 3. Survival time of mature spines. In this study we directly addressed the question in which phase of spinogenesis SynCAM 1 acts.

First, although we cannot fully exclude that SynCAM 1 has a spinogenic function, our data strongly suggest that SynCAM 1 does not directly initiate the formation of nascent protrusions, as we see a higher gain of nascent spines in the absence of SynCAM 1 and no change in spine gain upon SynCAM 1^flag^ overexpression. In contrast to the continuous overexpression, the induction of SynCAM 1^flag^ overexpression was accompanied by a transient increase in spine gain which returned to baseline within four weeks. Generally, this is explained by shifting the system from one steady state level to another; the observed increase in spine density requires a change in either the spine gain or the spine loss, or both. Mechanistically, elevating the SynCAM 1 level increases the likelihood of converting a nascent protrusions into a longer living spine which results in the observed spine gain. Accordingly, the number of filopodia-like structures decreases after inducing SynCAM 1^flag^ overexpression, supporting our idea that SynCAM 1 promotes the maturation of nascent protrusions. Consistent with this idea is the observation that turning on SynCAM 1^flag^ overexpression did not affect the stability of spines that were already present before starting SynCAM 1^flag^ overexpression. Taken together, our data indicate that SynCAM 1 has no direct spinogenic effect, but SynCAM 1 meditates the stabilization of nascent structures.

Second, SynCAM 1 works later in the process of spine formation by stabilizing nascent contacts. Although, it is difficult to identify the developmental stage of a dendritic protrusion, we considered that thin filopodia-like protrusions emerged recently and are developmentally young. The bigger fraction of thin filopodia-like protrusions in SynCAM 1 KO mice and smaller fraction in SynCAM 1^flag^ overexpressing animals suggest that these nascent structures are stabilized by SynCAM 1 and will mature further at a higher likelihood depending on SynCAM 1 abundance. The increased number of nascent spines in the absence of SynCAM 1 might be a result of a compensatory effect of the neuron failing to establish synaptic connections.

Third, the stabilizing action of SynCAM 1 is further reflected in the survival of spines that emerged between imaging sessions; spines which are at most three days old, but do not necessarily show a filopodia-like morphology. According to our idea that SynCAM 1 stabilizes nascent spines we see fewer of these nascent structures surviving in SynCAM 1 KO mice and more of them surviving in SynCAM 1^flag^ overexpressing mice. Moreover, the overall survival of spines also depends on SynCAM 1, demonstrating that SynCAM 1 not only stabilizes young synaptic contacts but also holds fully matured synapses together.

Surprisingly, the spine density did not return back to baseline, when SynCAM 1^flag^ overexpression was switched off months after induction. This indicates that the elevated spine density is maintained by other factors e.g. endogenous SynCAM 1, other adhesion molecules, synaptic activity, and homeostatic mechanisms than the overexpressed SynCAM 1^flag^. Similarly, it was shown by Hofer and colleagues that the spine density stays at an elevated level following the restoration of binocular vision after monocular deprivation^25^. In our previous study we showed that shutting down SynCAM 1^flag^ overexpression at P14 reversed the increase of mEPSC frequency within 2 weeks^19^. The cell types (hippocampal vs. cortical neurons), the time of overexpression (2 weeks vs 4 months), and the age of the animals (2–4 weeks vs 4 months) could account for this apparent difference. The prolonged overexpression of SynCAM 1^flag^ might be accompanied by axonal branching providing additional sites to form synaptic contacts, which preserve the increased spine density.

How stabilizes SynCAM 1 synaptic contacts? First, the transsynaptic interaction of SynCAM 1 with either SynCAM 1 or SynCAM 2 increases the adhesive forces between an axonal bouton and a dendritic spine. Second, besides the three extracellular Ig-like domains SynCAM proteins have an intracellular FERM and PDZ domain, which allow the interaction with other proteins^5^,^16^,^26^. Surprisingly little is known about interaction partners. The best studied protein binding to SynCAM 1 is Farp1, which binds to the FERM domain thereby regulating dendritic filopodial dynamics, increasing synapse number, and modulating spine morphology^26^. Mechanistically, Farp1 activates the GTPase Rac1 in spines downstream of SynCAM 1 clustering, and promotes F-actin assembly. Farp1 furthermore triggers a retrograde signal regulating active zone composition via SynCAM 1^26^. In summary, on the one hand SynCAM 1 is able to bind transsynaptically to SynCAM 1 and SynCAM 2 stabilizing spines, on the other hand SynCAM 1 engages intracellular protein interactions, which further promote the stability of synapses.

Only few adhesion molecules have been studied by chronic *in vivo* imaging. Recently it was shown that the neurexin family member CNTNAP2 does not affect the stability of stable spines, but stabilizes new spines^27^. Moreover, a Neurolign-3 mutation linked to autism spectrum disorders does not affect spine density but changes the turnover rate^28^. These results clearly distinguish SynCAM 1 from the function of the neurexin-neuroligin complex.

Many labs studied spine dynamics in the context of learning and memory by inducing plastic changes in the visual, auditory or barrel cortex, demonstrating the importance of spine dynamics for learning and memory^25^,^29^,^30^,^31^. We have demonstrated earlier that the SynCAM 1 knockout showed an enhanced performance in spatial learning, while in SynCAM 1^flag^ overexpressing mice failed the learning task^19^. Together with the presented findings, one could speculate that a higher synaptic dynamic improves adapting to new situations; future experiments are required to show this connection.

In summary, we demonstrate here that SynCAM 1 regulates spine number by stabilizing not only nascent protrusions but also mature spines.

## Materials and Methods

### Animal procedure

All animal procedures were performed in agreement with the European Union and German guidelines and were approved by the Government of North Rhine-Westphalia (Az. 84-02.04.2011.A108).

### Mouse Models

Previously described SynCAM 1^flag^ overexpressing mice^19^ were crossed to C57BL/6 mice expressing eGFP under the Thy1 promoter in a subset of cortical neurons (line GFP-M^32^). SynCAM 1^flag^ overexpressing is regulated by a tet-responsive element; the necessary tTA is expressed under the control of the CaMKII promotor^22^. To temporally control the overexpression of SynCAM 1^flag^ mice received doxycycline-containing water (1 g/l). Littermates lacking the SynCAM 1^flag^ transgene served as controls. Accordingly, SynCAM 1 Knockout mice^21^ were bred with GFP expressing mice and were compared to homozygotic littermates with normal SynCAM 1 expression. Animals were kept in a 12:12 h dark-light cycle with food and water access *ad libitum.* Mice were separately housed after the craniotomy.

### Surgery

Cranial surgery was performed on 12 week old mice as described elsewhere^33^. Briefly, mice were anesthetized with an. i.p. injection of Medetomidine (0.5 mg/kg, Cp-Pharma, Burgdorf, Germany), Midazolam (5 mg/kg kg, Ratiopharm, Ulm, Germany) and Fentanyl (0.05 mg/kg, Janssen-Cilag, Neuss, Germany). Caprofen (5 mg/kg, Pfizer, Berlin, Germany) was subcutaneous administered to reduce postsurgical pain. A 3–4 mm craniotomy was carefully done over the left hemisphere exposing the intact dura. The brain was covered with a 5 mm glass plate (Menzel, Braunschweig, Germany) and sealed with cyanoacrylate (UHU, Germany) and dental cement (Heraeus, Hanau, Germany). A small Delrin® bar with threaded holes was glued on the right hemisphere to fix the animal under the microscope. After surgery the anesthesia was reversed with Atipamezol (2.5 mg/kg, Orion Pharma, Hamburg, Germany), Flumazenil (0.5 mg/kg, Hameln Pharma plus, Hameln, Germany) and Naloxon (1.2 mg/kg, Ratiopharm, Ulm, Germany). The mice had 4 weeks to recover before the chronic imaging was started.

### *In vivo* imaging

For imaging the mice were lightly anesthetized with an i.p. injection of Ketamine (104 mg/kg, Medistar, Ascheberg, Germany) and Xylazine (8 mg/kg, Ceva, Düsseldorf, Germany) and head-fixed under the microscope. The body temperature was kept constant and the eyes protected from dehydration with eye ointment (Bayer, Leverkusen, Germany). We used a custom built two-photon microscope driven with a Ti:sapphire Laser (Chameleon Vision-S, Coherent, Santa Clara, CA) running at 910 nm for GFP excitation. The setup was controlled by ScanImage^34^. Image stacks of apical dendrites from Layer V pyramidal neurons were acquired with a water immersion 40× objective (LumPlanFl, Olympus, Hamburg, Germany). Each stack consisted of several sections with 1024 × 1024 pixels (0, 065 μm/pixel) and 0.3 μm z-spacing.

### Image analysis

Images were analyzed with a custom written software (MATLAB, Mathworks, Aachen, Germany). In total we analyzed 14723 individual spines present for up to eight different time points over a total dendritic length of 14.347 mm. Only dendrites of cells of which the soma was localized deeper than 350 μm measured from the cortex surface were analyzed. Scoring criteria were similar as described^33^. All protrusions emanating 0.4 μm laterally from the parent dendrite were identified as spines independent of their shape. We classified filopodia-like spines based on morphological criteria^35^; all protrusions with a minimum length of one dendrite diameter and no clear spine head (head to neck ratio < 1.1) were classified as filopodia-like spines. Spines were not classified in further categories. Spines were considered identical if the distinct position of their shaft on the dendrite did not differ for more than 0.5 μm between views. Spine scoring was conducted in the original 3D image stacks, whereas 2D image projections for each time point were used to label and track the individual spines. We categorized persistent new spines as spines that remained stable for at least 4 subsequent imaging days after their first appearance. For figure illustration we used Gaussian filtered maximum projections (ImageJ, NIH, USA) from single sections containing exclusively the information of the corresponding dendrite (Photoshop, Adobe, San Jose, CA).

### Immunoblotting

For Western blots cortex samples were sonicated and lysed in RIPA buffer. Samples were then separated using 8–10% SDS-PAGE and blotted on a polyvinylidene difluoride membrane (Merck-Millipore, Schwalbach, Germany). The following antibodies were used: Mouse anti-FLAG 1:1000 (clone M20, Sigma Aldrich, St. Louis, MO), mouse anti-actin 1:1000 (clone AC-40, Sigma Aldrich, St. Louis, MO) and HRP-linked horse anti-mouse 1:1000 (Cell Signaling Technology, Danvers, MA). Chemiluminescence signals were detected using a PeQlab Solo Imaging System (PEQLAB, Erlangen, Germany).

### Statistics

All imaging data were quantified blind to experimental conditions. Data were tested for normal distribution and statistical significance using Prism 5 (GraphPad, San Diego, CA). We used paired student’s t-tests to compare the means of different groups and unpaired t-tests for comparison of individual time points. Significance levels are denoted as follows: *p < 0.05; **p < 0.01; ***p < 0.001.

## Additional Information

**How to cite this article**: Körber, N. and Stein, V. *In vivo* imaging demonstrates dendritic spine stabilization by SynCAM 1. *Sci. Rep.* **6**, 24241; doi: 10.1038/srep24241 (2016).
